# Presumed Urinary Tract Infection in Patients Admitted with COVID-19: Are We Treating Too Much?

**DOI:** 10.3390/antibiotics10121493

**Published:** 2021-12-06

**Authors:** Johan Van Laethem, Stephanie C. M. Wuyts, Jan Pierreux, Lucie Seyler, Gil Verschelden, Thibault Depondt, Annelies Meuwissen, Patrick Lacor, Denis Piérard, Sabine D. Allard

**Affiliations:** 1 Department of Internal Medicine, Vrije Universiteit Brussel (VUB), Universitair Ziekenhuis Brussel, UZ Brussel, 1090 Brussels, Belgium; jan.pierreux@uzbrussel.be (J.P.); lucie.seyler@uzbrussel.be (L.S.); gil.verschelden@uzbrussel.be (G.V.); thibault.depondt@uzbrussel.be (T.D.); annelies.meuwissen@uzbrussel.be (A.M.); patrick.lacor@uzbrussel.be (P.L.); sabine.allard@uzbrussel.be (S.D.A.); 2 Hospital Pharmacy, Universitair Ziekenhuis Brussel (UZ Brussel), 1090 Brussels, Belgium; stephanie.wuyts@uzbrussel.be; 3 Research Group Clinical Pharmacology and Pharmacotherapy, Vrije Universiteit Brussel (VUB), 1090 Brussels, Belgium; 4 Microbiology Department, Universitair Ziekenhuis Brussel (UZ Brussel), 1090 Brussels, Belgium; denis.pierard@uzbrussel.be

**Keywords:** antimicrobial stewardship, antibiotics, bacterial respiratory tract infection, coinfection, COVID-19, superinfection

## Abstract

Despite the low rates of bacterial co-/superinfections in COVID-19 patients, antimicrobial drug use has been liberal since the start of the COVID-19 pandemic. Due to the low specificity of markers of bacterial co-/superinfection in the COVID-19 setting, overdiagnosis and antimicrobial overprescription have become widespread. A quantitative and qualitative evaluation of urinary tract infection (UTI) diagnoses and antimicrobial drug prescriptions for UTI diagnoses was performed in patients admitted to the COVID-19 ward of a university hospital between 17 March and 2 November 2020. A team of infectious disease specialists performed an appropriateness evaluation for every diagnosis of UTI and every antimicrobial drug prescription covering a UTI. A driver analysis was performed to identify factors increasing the odds of UTI (over)diagnosis. A total of 622 patients were included. UTI was present in 13% of included admissions, and in 12%, antimicrobials were initiated for a UTI diagnosis (0.71 daily defined doses (DDDs)/admission; 22% were scored as ‘appropriate’). An evaluation of UTI diagnoses by ID specialists revealed that of the 79 UTI diagnoses, 61% were classified as probable overdiagnosis related to the COVID-19 hospitalization. The following factors were associated with UTI overdiagnosis: physicians who are unfamiliar working in an internal medicine ward, urinary incontinence, mechanical ventilation and female sex. Antimicrobial stewardship teams should focus on diagnostic stewardship of UTIs, as UTI overdiagnosis seems to be highly prevalent in admitted COVID-19 patients.

## 1. Introduction

Antimicrobial resistance (AMR) is one of the biggest threats in the modern era of human and veterinary medicine. By 2050, it is expected that, without adequate action, mortality related to AMR could become the first cause of death worldwide, leading to a huge economic and humanitarian cost [1,2]. As the impact of the COVID-19 pandemic on AMR is a very complex and context-specific matter, further insights into the relationship between the human response towards the COVID-19 pandemic and the evolution of AMR are needed [3]. In order to counter antimicrobial overprescribing, antimicrobial stewardship teams need reliable data concerning antimicrobial prescriptions in admitted COVID-19 patients, as well as better diagnostic markers of bacterial co-/superinfection and enhanced decision support systems.

Since the start of the pandemic, different studies have highlighted disproportional antimicrobial use in COVID-19 patients despite low bacterial co- and superinfection rates. Reported rates vary from 4 to 19%, depending on the geographical setting of the study, the inclusion or exclusion of patients admitted to the intensive care unit and the used definitions of co-/superinfections [4,5,6,7,8,9]. Most investigators differentiate between bacterial respiratory co-/superinfection and nonrespiratory co-/superinfection. However, few evaluation data are available concerning the latter.

Among the most frequently occurring infections that are often overdiagnosed are urinary tract infections (UTIs) [10]. Various classical biomarkers used to detect bacterial infection, such as C-reactive protein, procalcitonin and the presence of fever, are not specific enough to diagnose co-/superinfections, as those markers may be altered by COVID-19 [11,12,13]. For example, in a retrospective cohort study including 3028 COVID-19 patients, Kubin et al. found that 51% of the reported coinfections were from urinary origin. However, the authors state that the cultures were not evaluated in the context of urinary symptoms [14]. In a retrospective study evaluating bacterial co- and superinfections in more than 64,000 COVID-19 patients, Baghdady et al. found unexpectedly high rates (18%) of bacterial coinfection. Of those, genitourinary infections were the most frequent (8.5% of the total population). However, according to the authors, this was probably an overestimation, as diagnoses were documented using diagnostic codes extracted from a federal database, and patient medical file review was not performed to confirm bacterial infection based on clinical criteria [9]. Additionally, urinary tract infections and asymptomatic bacteriuria (ASB) are frequently overdiagnosed and thus overtreated. Antimicrobial treatment initiation solely based on positive urinary sample results in the absence of clinical symptoms is not an appropriate practice [10,15]. The fatigue and stress associated with the COVID-19 pandemic, as well as the involvement in COVID-19 care of treating physicians unfamiliar with the management of urinary tract infections, could also have led to overdiagnosis of UTIs and inappropriate or unnecessary antimicrobial use for this indication. This underlines the importance of physician education and guidance by stewardship teams, especially in the rapidly changing and chaotic times of an infectious outbreak.

In this retrospective observational cohort study, our main goal was to evaluate the number of UTI overdiagnoses, as well as the amount and appropriateness of antimicrobial drug prescriptions started for a (presumed) UTI in admitted COVID-19 patients. Drivers associated with inappropriate UTI diagnoses were identified.

## 2. Materials and Methods

The study was conducted in the Universitair Ziekenhuis Brussel (UZ Brussel), a Belgian university hospital with a 721-bed capacity. All patients aged 18 years and older and admitted between 17 March and 2 November 2020, in the context of a SARS-CoV-2 infection, were eligible for inclusion. SARS-CoV-2 infection was diagnosed either by positive SARS-CoV-2 polymerase chain reaction (PCR) on nasopharyngeal swab or by clinical suspicion together with a strongly suggestive chest CT scan. SARS-CoV-2 variants were not yet prevalent in Belgium during the inclusion period. Patients admitted for less than 24 h were excluded.

The primary objective of this study was to retrospectively evaluate the rate of UTI overdiagnosis, expressed as the percentage of UTIs, which should not have been withheld according to the local, national and international diagnostic guidelines, as well as to identify drivers associated with misdiagnosed ‘UTI’. The data were extracted from the patient electronic medical files following a structured registration method. These data included demographics, comorbidities, symptoms, laboratory results, prognostic factors (quantified sequential organ failure assessment (qSOFA) score at admission, amount of oxygen needed, mechanical ventilation, ICU hospitalization, survival), microbiological data, specific risk factors for (complicated) UTI or asymptomatic bacteriuria (including the presence of external urinary catheters, urinary incontinence, functional or anatomical urinary tract disorders, living in a nursing home, ‘decreased autonomy’) and data related to antimicrobial prescriptions. UTI diagnoses were classified according to anatomical criteria (cystitis, prostatitis, pyelonephritis, catheter-associated urinary tract infection (CAUTI)) and labeled as ‘complicated’ or ‘uncomplicated’. Used definitions for fever, calculation of oxygen need, ‘complicated UTI’, immune suppression and ‘decreased autonomy’, as well as the method for microbiological identification and antimicrobial susceptibility testing, can be found in the Supplementary Materials [16,17]. Urinary culture results were only documented if the diagnosis and/or therapy of a UTI diagnosis was based on its result. For all included patients, an infectious disease specialist carefully screened for documented UTI diagnoses by consulting the hospitalization letters and notes in the UZ Brussel electronic hospital information system during the hospitalization on a COVID-19/ICU ward. Each UTI diagnosis and antimicrobial prescription (belonging to the ‘Anatomical Therapeutic Chemical’ classification group J01 and J02 [18]) in the context of this UTI diagnosis was evaluated by a panel of maximum three infectious diseases (ID) specialists, in which two ID specialists evaluated independently from each other the appropriateness of each UTI diagnosis and antibiotic prescription; in the case of disagreement in the evaluation of appropriateness, a third ID specialist served as a mediator to come to a consensus with the two other ID specialists. UTI diagnoses were classified as ‘appropriate’ or ‘inappropriate’. ‘Inappropriate’ diagnoses were divided as ‘not meeting the diagnostic criteria of UTI (‘overdiagnosis’, e.g., asymptomatic bacteriuria)’ or ‘wrong diagnostic classification’ (e.g., pyelonephritis instead of cystitis). For the UTI overdiagnoses, a differentiation was made between ‘probably related to COVID-19 admission’ and ‘probably unrelated to COVID-19 admission’ (e.g., UTI in a patient admitted for another reason than COVID-19; for definitions: see Supplementary Materials. For every evaluated diagnosis, the ID specialists were asked to declare how certain they were of their decision (not sure, pretty sure or certain).

The secondary endpoint was the degree of antimicrobial prescriptions appropriateness for (presumed) UTI diagnoses. Antimicrobial prescriptions in the context of UTIs were analyzed in terms of daily defined doses (DDDs) for every prescribed antimicrobial class, as proposed by the WHO [18]. The appropriateness of antimicrobial prescription was determined by the same ID specialist panel as mentioned above and categorized as ‘appropriate’, ‘inappropriate’, ‘unnecessary’ or ‘suboptimal’. A maximum of three different reasons to initiate a certain antimicrobial course were registered (see Supplementary Materials).

The following variables of interest were included in the analysis to determine risk factors associated with UTI overdiagnosis: age, sex, decreased autonomy, presence of external urinary catheters, urinary incontinence, functional or anatomical urinary tract disorders, living in a nursing home, the presence of a chronic neurological disease (including sequellae), Charlson Comorbidity Index (CCI) [19], dementia, the medical specialty of the treating physician, the C-reactive protein (CRP) value, need for ICU admission, lowest SpO2/FiO2 rate during hospitalization, fever at admission, duration of hospitalization on the COVID-19 or ICU ward, need for mechanical ventilation and the absolute count of pyuria.

Statistical analyses were performed using the statistical software ‘IBM SPSS statistics version 28.0.0.0′. Descriptive statistics were applied to characterize the cohort, reporting percentages and medians with interquartile ranges (IQRs) for all included patients. The DDDs were calculated for each UTI antimicrobial drug in general and as a function of the appropriateness. In a logistic regression model, to identify drivers associated with (inappropriate) UTI diagnoses, a backwards logistic regression was performed. Multicollinearity among variables was taken care of by verifying the variance inflation factors (VIFs) of each driver (VIF < 5). Only significant variables were retained in the final model. Results with a value of *p* < 0.05 were considered statistically significant.

### Compliance with Ethics Guidelines

This study was approved by the local ethics committee (Commissie Medische Ethiek, UZ Brussel) prior to data collection (B.U.N. 1432021000630) and was carried out in accordance with the ethical principles for medical research involving human subjects established by the Declaration of Helsinki, protecting the privacy of all participants, as well as the confidentiality of their personal information. Because of the retrospective nature of the study, a waiver for the informed consent form was obtained.

## 3. Results

### 3.1. Patient and Disease Characteristics

**Figure 1:**
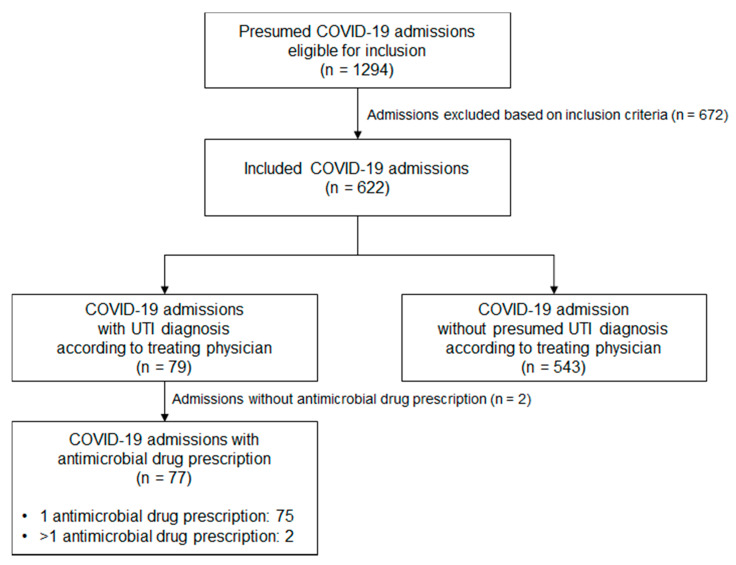
Flow chart of the included admissions with a COVID-19 diagnosis admitted to the COVID-19 ward/ICU.

A total of 1294 admissions were eligible for inclusion. After exclusion of patients with a rejected diagnosis of COVID-19 or a hospital stay less than 24 h, 622 admissions were included in this retrospective cohort. In these 622 admissions, the treating physician diagnosed a UTI in 79 (13%) of them (Figure 1).

Demographics, symptoms and comorbidities predisposing for poor COVID-19 outcome [7] are represented in Table 1. Risk factors for asymptomatic bacteriuria or (complicated) urinary tract infection [20] were present in 46% of this retrospective cohort, namely: 31% of included patients had decreased autonomy, 15% were urinary incontinent at admission, 14% had an active or passive neurological disease with sequelae at admission, 13% lived in a nursing home, 12% had an anatomical urinary tract pathology, 11% was known with a cognitive disorder, 10% had a functional urinary tract pathology and 3% had a chronic urinary catheter at admission. Mechanical ventilation was indicated in 7% of all included patients. The overall mortality rate was 8% (Table 1).

At least one urinary sample was collected for bacteriological investigation in 410/622 (66%) inclusions. The median pyuria count was 158 and 636/µL for, respectively, all inclusions and for those with diagnosis of UTI by the treating physician. Urinary cultures of the 79 patients with UTI diagnosis were positive in all of them, with isolation of a total of 91 microorganisms. *Enterobacterales* (67/91, 74%) were by far the most commonly isolated bacteria, with *Escherichia coli* as the main representative (48/91, 53%). Four extended spectrum beta-lactamase (ESBL)-producing *Escherichia coli* were recorded (Supplementary Materials Table S4). There were no carbapenemase-producing *Enterobacterales* (CPE). Only two urinary cultures positive for fungi were identified as UTI: one with *Candida albicans*, as well as *Candida glabrata* in the same culture, and one with *Candida tropicalis*. Further specification of the isolated urinary pathogens is available in Tables S2 and S3 in the Supplementary Materials. A bacteremia of urinary origin was identified in eight patients, of which five *Escherichia coli* bacteremias. Only one fungaemia with *Candida tropicalis* was detected. Sepsis of urinary origin was withheld in 11/79 (14%) of the patients with diagnosis of UTI.

### 3.2. UTI Diagnoses

Of the 79 patients with at least one UTI diagnosis by the treating physician, details regarding the anatomical classification and the classification, including complicating factors, were not provided in, respectively, 31/79 (39%) and 22 (28%) of the patients. For the remaining 48 patients regarding the anatomical classification, pyelonephritis (19%), cystitis (18%) and prostatitis (14%) were more frequently diagnosed than catheter-associated urinary tract infections (10%). A complicated UTI was diagnosed in 35/79 (44%) of the patients (Supplementary Materials Table S5). In the 79 patients with UTI diagnosis by the treating physician, a total of 77 (97%) antimicrobial courses were initiated. For two patients, a diagnosis of UTI was mentioned in the hospitalization letter but not treated due to asymptomatic bacteriuria. A total of 143 motivations (median: 2 per included admission, IQR: 1) for antimicrobial therapy were mentioned in 78/79 (99%) patient files, with pyuria and bacteriuria (38%), fever (15%) and an inflammatory syndrome (8%) as main reasons (Supplementary Materials Table S6).

**Figure 2:**
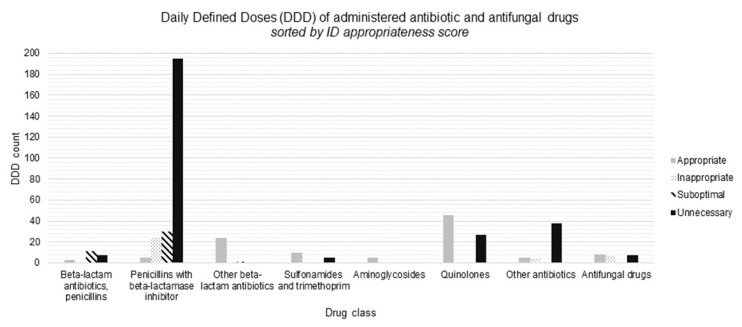
Appropriateness of antimicrobial consumption in diagnosed UTIs. X-axis: different classes of antimicrobials including antifungals. Y-axis: absolute count of daily defined doses (DDDs) administered for UTI diagnoses by the treating physician.

Penicillins with a beta-lactamase inhibitor were administered most frequently (58% of all DDDs). Details on antimicrobial consumption are shown in Figure 2 and Table 2. The median lag time from the first day of hospitalization in a COVID-19 ward or ICU until the start of the first dose of antimicrobial for UTI diagnosis was 5 days (IQR: 6).

### 3.3. Evaluation of UTI Diagnoses and Antimicrobial Prescriptions

In the systematic evaluation of the diagnostic and antimicrobial’s appropriateness, the two ID specialists disagreed in, respectively, 18% (14/79) and 35% (27/77) of patients. A final consensus was reached for all patients. Of all UTI diagnoses, 61% (48/79) were scored as ‘overdiagnosed’, and all of those were scored as ‘probably related to COVID-19′, except one, which was classified as ‘probably not related to COVID-19′. There were no UTI diagnoses classified as ‘wrong diagnostic classification’. Only 22% of antimicrobial consumption was evaluated as appropriate. The majority (62%) of all prescribed DDDs were considered unnecessary, while 6% and 10% of the DDDs were labeled, respectively, inappropriate and suboptimal. The intravenous rate of prescribed antimicrobial DDDs for UTI was 48%.

### 3.4. Associated Drivers with UTI (over) Diagnosis

Logistic regression analysis of potential drivers associated with the diagnosis of UTI by the treating physician identified the following drivers: need for mechanical ventilation (odds ratio (OR): 10.42; 95% confidence interval (CI): 3.15–34.50), the presence of an anatomical or functional urinary tract pathology (OR: 5.11; 95% CI: 2.31–11.32), the presence of an active or passive chronic neurological disease with sequelae (OR: 2.95; 95% CI: 1.30–6.69), fever at admission (OR: 2.57; 95% CI: 1.18–5.60), older age (per increase of 1 year, OR: 1.03; 95% CI: 1.00–1.06) and CRP (per rise of 1 mg/dL, OR: 1.01; 95% CI: 1.00–1.01). Male sex was identified as an inversely correlated variable (OR: 0.15; 95% CI: 0.06–0.38; Table 3). The following variables were identified as drivers of a UTI overdiagnosis: a UTI diagnosis made by a physician who was unfamiliar with the internal medicine ward (OR: 34.48; 95% CI: 10.22–116.29), urinary incontinence (OR: 8.78; 95% CI: 3.84–20.05) and need for mechanical ventilation (OR: 3.70; 95% CI: 1.07–12.81). Male sex was again retained as an inversely correlated variable (OR: 0.15; 95% CI: 0.06–0.38; Table 4).

## 4. Discussion

In this retrospective study, we found high rates (61%) of inappropriate UTI diagnoses, which resulted in a significant proportion of unnecessary antimicrobial use for UTIs diagnosed by the treating physician. Various studies have reported on incidence rates of nonrespiratory bacterial co-/superinfection in COVID-19 admissions. However, a specification of the type of infection is seldom made. Moreover, the few studies that do report on UTIs are solely based on microbiological diagnostic criteria, neglecting the importance of clinical diagnostic criteria [18]. Appropriateness studies focusing on UTI diagnoses and treatment are scarce, especially in the COVID-19 setting [6,9,14,21,22]. Reyes et al. came with a warning concerning probable overdiagnosis and overtreatment of asymptomatic bacteriuria in COVID-19 patients, as they cited the Infectious Disease Society of America (IDSA), which recommends ‘the assessment for other causes and watchful observation of older patients with functional and/or cognitive impairment with bacteriuria and without local genitourinary symptoms rather than immediate antimicrobial treatment’. Although the IDSA guidelines do not consider the presence of systemic signs of infection, which are prevalent in COVID-19 patients, Reyes et al. still recommend a meticulous evaluation and conservative approach of bacteriuria in the absence of genitourinary symptoms, particularly in delirious COVID-19 patients [10,23].

More than three-quarters of prescribed antimicrobials were classified as not appropriate. This is in line with several pre-COVID-19 studies. Duane et al. concluded that less than 40% of outpatient antimicrobial prescriptions for (presumed) UTI were in accordance with first-line treatment recommendations. Studies in long-term facility care settings reported that antimicrobial treatment initiation was appropriate in only 49% to 63% of patients [24,25,26,27,28,29]. Appropriate antimicrobial use in patients with complicated UTI seems to be associated with a reduced length of hospital stay, improved patient outcomes and lower healthcare costs. Therefore, investing in antimicrobial stewardship interventions directed towards appropriate UTI diagnosis and treatment, particularly in a COVID-19 setting, is crucial to promote judicious antimicrobial use [30]. Our data could not be compared with other institutions, as, to our knowledge, we are the first to generate data on diagnosis of UTIs in a COVID-19 setting. Nevertheless, our antibiotic consumption data are very similar to those of a recent prospective antimicrobial evaluation study of (presumed) bacterial respiratory tract co-/superinfection in a similar setting (8.19 DDDs/100 hospital bed days in our retrospective study versus 8.92 DDDs/100 hospital bed days in the bacterial respiratory co-/superinfection evaluation study) [31]. Looking at the antimicrobial appropriateness evaluation, Van Laethem et al. showed a higher rate of appropriate use of antimicrobials (39% versus 22% in this study), which is in line with a prospective appropriateness study of van Buul et al., in which decisions around antimicrobial initiation for UTI were less often appropriate compared to respiratory indications [32]. Olafsson et al. described antimicrobial prescription numbers of 1.74 DDDs/inhabitant/100 days for UTI diagnoses in an outpatient setting. However, it is difficult to compare this data with ours, as the study took place in a totally different setting, exclusively included women and was performed before the COVID-19 pandemic [33].

Penicillins with a beta-lactamase inhibitor were prescribed most frequently (4.73 DDDs/100 bed days) and represented 70% of all unnecessary DDDs. This is probably due to the first-line empiric use of penicillins with a beta-lactamase inhibitor for a lot of infectious indications in our setting. Conversely, national and local guidelines use third-generation cephalosporins as first-choice empiric treatment for pyelonephritis and complicated UTIs, due to antimicrobial resistance rates of *Escherichia coli* towards penicillins with beta-lactams exceeding 20% in Belgium [34]. *Escherichia coli* represented more than half (53%) of the isolated microorganisms causing UTIs diagnosed by the treating physician, which is in line with previous research [35,36]. *Staphylococcus aureus* and *Staphylococcus saprophyticus* infections were not identified in this study, which can be explained by their low representation in UTI and the older age of patients in our study population [37,38]. Rates of bacteremia associated with UTI vary across studies and range between <10 and 50%, depending on the included patient population. In our study population, the bacteremia rate was 10% in the patients in whom UTI was diagnosed [35,39,40].

Urinary incontinence was shown to be a driver of UTI overdiagnosis. This was expected as urinary incontinence is known to be a risk factor of ASB [41]. Moreover, Warren et al. found ASB rates up to 50% in elderly institutionalized persons [42]. Although we identified older age as a driver of UTI diagnosis, it was not a driver of UTI overdiagnosis. This could be explained by the lower rates of institutionalized patients in this study (13% for all included admissions and 34% of included UTI diagnoses) compared to other studies [41,43,44,45]. Indeed, age in itself is a known risk factor for the development of UTI due to immune paresis, lower urinary output and higher rates of functional and anatomical urinary tract pathologies, but not necessarily directly related to UTI overdiagnosis. This last entity seems to be more dependent on the functional status of the patient. UTIs are quite rare in the ICU setting [22]. Therefore, the fact that mechanical ventilation was identified as a risk factor for UTI overdiagnosis is probably related to the overdiagnosis and overtreatment of ASB in patients with bacterial and/or fungal colonization associated with long-term catheterization. Moreover, the frequent persisting fever and fluctuating inflammatory markers levels in often hemodynamically unstable patients might have led to overtreatment. Additionally, physicians less experienced in internal medicine were employed on COVID-19 wards, which added to the risk of unconscious use of antimicrobials [46,47,48]. Our study indeed shows that physicians who were not familiar with working on an internal medicine/ICU ward dramatically increased the odds of UTI overdiagnosis. As UTIs are less frequently encountered in male patients, due to anatomical and physiological differences, it is understandable that male sex significantly decreased the odds of UTI diagnosis and overdiagnosis in our setting [49,50,51]. Moreover, as male sex is a complicating factor of UTIs, ID specialists might have been more careful before labeling diagnosed UTIs by the treating physician as overdiagnosis. It should be noted that the drivers associated with UTI (over)diagnosis do not necessarily imply a causal relationship.

This study had several limitations, primarily due to its retrospective character. First, since the appropriateness scoring was performed based on retrospective chart review, subtleties, which are needed to make a judicious diagnosis might have been missed. This is illustrated by insufficient documentation regarding UTI classification in 39% of cases. Moreover, ID specialists are also subject to uncertainties when diagnosing bacterial co-/superinfections in COVID-19 patients, as clinical parameters, including inflammatory markers, fever and procalcitonin, are often not specific in this setting [11,12,13,52]. However, the low disagreement rates between the two ID physicians, who scored independently from each other, illustrated that the majority of the scoring process was straightforward. Moreover, most of the diagnostic scorings were labeled as ‘pretty sure’ or ‘certain’. Second, antimicrobial prescriptions were exclusively reviewed in the setting of UTI diagnoses. Thus, certain antimicrobial prescriptions could have been labeled as ‘not appropriate’ for a UTI diagnosis while being appropriate for another concomitant infectious disease. Finally, since the inclusion period was substantially long, a possible learning curve effect could have occurred. However, a decrease in (inappropriate) antimicrobial prescriptions over time was not documented for the purpose of this study.

To our surprise, our study results could not be compared to any other in-hospital UTI antimicrobial consumption study. This is why similar (prospective) antimicrobial evaluation studies are needed to enhance the awareness of overdiagnosis and overtreatment of UTIs and also to understand the factors that drive inappropriate antimicrobial use.

## 5. Conclusions

Despite the evidence of low rates of bacterial co-/superinfections in COVID-19 patients, UTI diagnoses seem to be inappropriate in a significant proportion of admissions, leading to unnecessary antimicrobial use. Previously reported rates of bacterial co-/superinfections in COVID-19 patients should be interpreted cautiously, as included diagnoses are often exclusively based on microbiology without qualitative assessment of the diagnostic and antimicrobial prescribing process. Complementary studies are needed to expand the knowledge concerning the true incidence of bacterial and fungal co-/superinfections in general but also explicitly for the different subtypes of nonrespiratory infections. In order to counter antimicrobial resistance and other harmful consequences of inappropriate antimicrobial use, antimicrobial stewardship teams should be granted sufficient resources to promote judicious antimicrobial use, especially in the COVID-19 era.

## Figures and Tables

**Table 1:** Patient characteristics.

	All Admissions (*n* = 622)	Admissions with UTI Diagnosis by the Treating Physician (*n* = 79)
Demographics		
Age (years); (median, IQR *)	63 (15)	77 (16)
Gender (male); (*n*, %)	359 (58)	22 (28)
BMI ** (kg/m^2^); (median, IQR)	27 (6)	25 (7)
COVID-19 diagnosis (*n* (%));		
• PCR ***	556 (89)	72 (91)
• Clinical Diagnosis	66 (11)	7 (9)
Length of stay (median, IQR)	7 (6)	10 (10)
COVID-19 related symptoms at admission (*n*, %)		
Cough	363 (58)	29 (37)
Fever or history of fever	421 (68)	52 (67)
Dyspnea	358 (58)	27 (35)
Thoracic pain	142 (23)	7 (9)
Laboratory findings (median, IQR; except for lymphopenia)		
White blood cell count (/mm^3^)	6600 (4600)	7800 (7300)
Neutrophil count (/mm^3^)	4845 (4188)	5540 (5867)
Lymphocyte count (/mm^3^)	1010 (633)	1010 (645)
Ferritin (mcg/L)	580 (869)	465 (687)
CRP ^†^(mg/dL)	75 (127)	72 (129)
Comorbidities		
CCI ^‡^ (median, IQR)	1 (3)	2 (4)
Diabetes mellitus (*n*, %)	162 (26)	26 (33)
Pre-existing pulmonary disease (*n*, %)	79 (13)	13 (17)
Ischemic/congestive heart disease (*n*, %)	72 (12)	11 (14)
Other variables, possibly related to ASB ^☩^ or UTI ^§^		
Urinary incontinence (*n*, %)	91 (15)	36 (46)
Presence of a chronic urinary catheter (*n*, %)	16 (3)	6 (8)
Living in a nursing home (*n*, %)	79 (13)	27 (34)
Decreased autonomy (*n*, %)	194 (31)	54 (68)
Anatomical urinary tract pathology	77 (12)	19 (24)
Functional urinary tract pathology	63 (10)	23 (29)
Active neurological disease or passive with sequelae (*n*, %)	87 (14)	25 (32)
Cognitive disorder (*n*, %)	68 (11)	20 (25)
Active immune suppression (*n*, %)	84 (14)	13 (17)
Prognostic factors		
qSOFA score at admission (median, IQR)		
0	303 (49)	33 (43)
1	272 (44)	36 (47)
2	28 (5)	7 (9)
3	2 (0)	1 (1)
ICU admission (*n*, %)	126 (20)	19 (24)
Mechanical ventilation need (*n*, %)	46 (7)	8 (10)
(SpO2/FiO2 × 100) min ^X^ (median, IQR)	296 (190)	284 (222)
Mortality (*n*, %)	23 (8)	14 (18)

* IQR: interquartile range; ** BMI: body mass index; *** PCR: polymerase chain reaction; ^†^ CRP: C-reactive protein; ^☩^ ASB: asymptomatic bacteriuria; ^§^ UTI: urinary tract infection; ^‡^ CCI: Charlson Comorbidity Index; ^X^ SpO2/FiO2 × 100 min = the lowest value of the SpO2/FiO2 rate during the total stay on a COVID-19 ward or in the ICU.

**Table 2:** Appropriateness of antimicrobial consumption in UTI, per antimicrobial class.

Type of Antimicrobial Drugs	Appropriate DDDs	Inappropriate DDDs	Suboptimal DDDs	Unnecessary DDDs	Total DDDs
Beta-lactam antibiotics, penicillins	2.3	0	11.5	7.7	21.5
Penicillins with beta-lactamase inhibitor	5.2	23.5	30.2	195	253.9
Other beta-lactam antibiotics	24	0	0.7	0	24.7
Sulfonamides and trimethoprim	10	0	0	5	15
Aminoglycosides	4.9	0	0	0	4.9
Quinolones	45.5	0.5	0	26.8	72.8
Other antibiotics	5	3.8	0	37.5	46.3
Antifungal drugs	8	7	0	7	22

DDD: daily defined dose.

**Table 3:** Mixed-effects logistic regression analysis of potential drivers associated with the (presumed) diagnosis of UTI.

Variable	OR (95% Confidence Interval)	*p*-Value
Diagnosis of UTI ^§^ (*n* = 79)		
Age (per increase of 1 year)	1.03 (1.00–1.06)	0.043 *
Male sex	0.24 (0.11–0.53)	<0.001
Active cerebrovascular disease or sequelae		
No	Ref.	
Yes	2.95 (1.30–6.69)	0.009 *
C-reactive protein level at admission (per rise of 1 mg/dL)	1.01 (1.00–1.01)	0.007 *
Fever at admission	2.57 (1.18–5.60)	0.018 *
Anatomical or functional urinary tract pathology	5.11 (2.31–11.32)	<0.001
Mechanical ventilation need	10.42 (3.15–34.50)	<0.001

* *p* < 0.05 is considered statistically significant; § UTI: Urinary tract infection.

**Table 4:** Mixed-effects logistic regression analysis of potential drivers associated with overdiagnosis of UTI.

Variable	OR (95% Confidence Interval)	*p*-Value
Overdiagnosis of UTI ^§^ (*n* = 40)		
Male sex	0.15 (0.06–0.38)	<0.001
Urinary incontinence	8.78 (3.84–20.05)	<0.001
Physician unfamiliar with work in an internal medicine/ICU ward	34.48 (10.22–116.29)	<0.001
Mechanical ventilation need	3.70 (1.07–12.81)	0.039

*p* < 0.05 is considered statistically significant; § UTI: urinary tract infection.

## Data Availability

The datasets generated during and/or analyzed during the current study are available from the corresponding author on reasonable request.

## Supplementary Materials

The following are available online at https://www.mdpi.com/article/10.3390/antibiotics10121493/s1, Table S1: Definitions used to score appropriateness of antimicrobial prescriptions, Table S2: Microbiological data part 1/2, Table S3: Microbiological data part 2/2, Table S4: Specification and antibiograms of extended-spectrum beta-lactamase (ESBL) producing microorganisms, Table S5: classification of UTI according to anatomical criteria or complicating factors, Table S6: Reasons mentioned for the initiation of antimicrobial treatment.
