# Supplementary material for: Presumed Urinary Tract Infection in Patients Admitted with COVID-19: Are We Treating Too Much?
Source: Antibiotics (Basel). 2021 Dec 6;10(12):1493. doi: 10.3390/antibiotics10121493 (PMC8698875; doi:10.3390/antibiotics10121493)
Supplement: Supplementary file 1 [file antibiotics-10-01493-s001.zip › antibiotics-1481077-supplementary.pdf]

Supplementary material

Supplementary Material 1: Applied definitions in patient data collection and used methods for microbiological identification and susceptibility testing

-Fever was defined as a tympanic temperature above 38°C.

-Need for supplemental oxygen was documented by calculating the pulsed finger saturation to fraction of inspired oxygen ratio (SpO2/FiO2).

-'Complicated UTI': All UTIs that are not defined as uncomplicated. Meaning in a narrower sense UTIs in a patient with an increased risk of a complicated course: i.e. all men, pregnant women, patients with relevant anatomical or functional abnormalities of the urinary tract, indwelling urinary catheters, renal diseases, and/or with other concomitant immunocompromising diseases.

-decreased autonomy: classified as being dependent in one of the following six functions according to the Katz scale, namely, bathing, dressing, going to toilet, transferring, continence, and feeding (ref. Katz et al).

-Active immune suppression: active solid or haematological cancer, recent chemotherapy or immunotherapy (during the last 6 months), autoimmune disease, autoinflammatory disease, rheumatoid arthritis, inflammatory bowel disease, primary immune deficiency, intake of 'biologicals', intake of high doses of corticoids or other medications that can diminish the immune system.

-Method used for microbiological identification: identification was performed by matrix-assisted laser desorption ionization time of flight mass spectrometry (MALDI-TOF MS) using a Microflex LT mass spectrometer with MALDI Biotyper 3.0 software and Reference Library 3.3.2.0 (Bruker Daltonik GmbH, Bremen, Germany).

-Method used for antimicrobial susceptibility testing: Disk diffusion tests were performed according to The European Committee on Antimicrobial Susceptibility Testing (EUCAST) on Mueller-Hilton agar (I2A, Montpellier, France). The Mueller-Hilton agar plates were incubated during 24 hours at 37°C. After incubation, the zone of inhibition was measured by SIR scan® (I2A, Montpellier, France).The algorithm for phenotypic detection of ESBL from EUCAST was used to interpret the disk diffusion diameters.

Supplementary Material 2: Definitions used to score appropriateness UTI diagnoses

-appropriate diagnosis: every diagnosis meeting the best available evidence at this time, and/or meeting the local, regional, national and/or international guidelines. In cases of conflicting local/national guidelines with international guidelines (due to a different setting and flora), local guidelines were followed.

-inappropriate diagnosis: every diagnosis not meeting the definition of appropriate diagnosis (e.g. asymptomatic bacteriuria in a non-pregnant woman diagnosed as UTI)

-not meeting the diagnostic criteria of any UTI (overdiagnosis): every inappropriate diagnoses which can be labelled as an unnecessary diagnosis (same example as above).

-wrong diagnostic classification: every inappropriate diagnosis which can be labelled as a UTI, but for which the anatomical classification is incorrect. (e.g. 'cystitis' instead of 'pyelonephritis').

-overdiagnosis probably related to COVID-19 admission: every admission, primarily due to COVID-19 disease or its consequences (eg. fever), which probably led to an overdiagnosis of a UTI. Admissions due to any other reason than COVID-19 (eg. appendicitis) with a fortuitous diagnosis of COVID-19 and without signs or symptoms related to COVID-19 that could mimic UTI (fever, high inflammatory markers…) will be classified as 'probably not related to COVID-19 admission'.

Supplementary Material 3: Definitions used to score appropriateness of antimicrobial prescriptions

Table S1. Definitions used to score appropriateness of antimicrobial prescriptions

Appropriate	The use of antimicrobials in the setting of an established or empirical infection that cannot be improved in one of the following categories: Indication, drug choice, drug route and drug dose.
Inappropriate	The use of antimicrobials in the setting of established infection to which the pathogen is resistant, the use of antimicrobials not recommended in treatment guidelines or absolute/relative contraindications (prolonged QTc, type 1 hypersensitivity, important interactions...)
Suboptimal	The use of antimicrobials in the setting of established infection that can be improved in one of the following categories: drug choice, drug route and/or drug dose.
Unnecessary	The use of antimicrobials for nonbacterial infections, days of therapy beyond the indicated duration of therapy without any clinical reason for lengthened course, use of redundant antimicrobial therapy, continuation of empiric broad-spectrum therapy when cultures have revealed the infecting pathogen.

From Spivak ES, Cosgrove SE, Srinivasan A. Measuring Appropriate Antimicrobial Use: Attempts at Opening the Black Box. Clin Infect Dis. 2016;63(12):1639-44.

Supplementary Material 4: Microbiological data

Table S2. Microbiological data part 1/2

	All included admissions (n= 622)	Included admissions with UTI[§] diagnosis by the treating physician (n = 79)
**General data (n, % per admission)**		
At least one urinary sample	410 (66)	79 (100)
Pyuria (median, IQR[X])	158 (643)	636 (743)

At least one prescription of antimicrobials for (presumed) UTI	77 (12)	77 (97)
Bacteremia of urinary origin	8 (1)	8 (10)

§ UTI: Urinary tract infection; Ⅹ IQR: Interquartile range;

Table S3. Microbiological data part 2/2

**Microbiological specification of urinary cultures in admissions with (presumed) UTI diagnosis (n,%)**	**91 (100)**
**Aerobic, gram positive cocci**	**11 (12)**
Streptococcus spp.	1 (1)
Aerococcus spp.	4 (4)
Staphylococcus haemolyticus	1 (1)
Enterococcus faecium	1 (1)
Enterococcus faecalis	4 (4)
**Aerobic, gram negative bacilli, *Enterobacterales***	**67 (74)**
ESBL[¥] in urinary tract sample*	4 (4)
CPE[ø] in urinary tract sample	0 (0)
Klebsiella pneumoniae	9 (10)
Escherichia coli	48 (53)
Proteus spp.	4 (4)
Enterobacter cloacae	1 (1)
Other	1 (1)
**Aerobic, gram negative bacilli, non-fermenter**	**4 (4)**
Pseudomonas aeruginosa	4 (4)
**Anaerobic, gram positive bacilli**	**1 (1)**
Lactobacillus spp.	1 (1)
**Positive culture without specification**	**5 (5)**
Mixed culture	5 (5)
**Fungi, yeasts**	**3 (3)**
Candida albicans	1 (1)
Candida glabrata	1 (1)
Candida tropicalis	1 (1)

§ UTI: Urinary tract infection; Ⅹ IQR: Interquartile range; **¥** ESBL: Extended Spectrum Beta-Lactamase; **ø** CPE: Carbapenemase producing Enterobacterales; * For ESBL specification and antibiograms, see Table S4.

Supplementary Material 5: Specification and antibiograms of extended spectrum beta-lactamase (ESBL) producing micro-organisms.

Table S4. Specification and antibiograms of extended spectrum beta-lactamase (ESBL) producing *Escherichia coli*.

**ESBL *Escherichia coli***	**Susceptible**	**Intermediate susceptible**	**Resistant**
Isolate A	pip-tazo, temo, mero, amika, fosfo	none	amoxi-clav, cefadro, cefur, ceftri, cefep, aztreo, cipro, trim/smx
Isolate B	pip-tazo, temo, mero, amika, trim/smx, fosfo	cefep, aztreo	ampi, amoxi-clav, cefadro, cefur, ceftri, trim/smx
Isolate C	cefur, ceftri, temo, mero, aztreo, cipro, amika, trim/smx, fosfo	cefep	ampi, amoxi-clav, pip-tazo, cefadro
Isolate D	Pip-tazo, mero, amika, trim/smx, fosfo	none	ampi, amoxi-clav, cefadro, cefur, ceftri, cefep, temo, aztreo, cipro

ESBL: Extended Spectrum Beta-Lactamase; ampi: ampicillin; amoxi-clav: amoxicillin-clavulanate; pip-tazo: piperacillin-tazobactam; temo: temocillin; mero: meropenem; amika: amikacin; fosfo: fosfomycin; cefadro: cefadroxil; cefur: cefuroxime; ceftri: ceftriaxone; cefep: cefepime; aztreo: aztreonam; cipro: ciprofloxacin; trim/smx: trimethoprim/sulfamethoxazole

Supplementary Material 6: classification of UTI according to anatomical criteria or complicating factors

Table S5. classification of UTI according to anatomical criteria or complicating factors

**Total of UTIs (n, %)**	**79 (100)**
**Anatomical classification**	79 (100)
Cystitis	14 (18)
Pyelonephritis	15 (19)
Prostatitis	11 (14)
Catheter associated urinary tract infection	8 (10)
Not specified	31 (39)
**Complicating factors**	79 (100)
Uncomplicated	22 (28)
Complicated	35 (44)
Not specified	22 (28)

Supplementary Material 7 : Reasons mentioned for the initiation of an antimicrobial treatment

Table S6. Reasons mentioned for the initiation of an antimicrobial treatment*

**Total amount of reasons (n, %)**	**143 (100)**
Not mentioned	3 (2)
Lower urinary tract symptoms	7 (5)
Fever	21 (15)
Inflammatory syndrome	12 (8)
Pyuria	5 (3)
Bacteriuria	2 (1)
Pyuria and bacteriuria	54 (38)
Bad smelling or cloudy urine	4 (3)
Confusion	3 (2)
Radiological signs of UTI§	2 (1)
Positive urinary culture with a yeast	2 (1)
Urinary retention	7 (5)
Sepsis	7 (5)
Haematuria	3 (2)
Pregnancy and positive urinary culture	0 (0)
Positive costolumbar point	1 (1)
Other	10 (7)

*Up to 3 reasons per antimicrobial treatment; § UTI: Urinary tract infection
